# Proximal Fibular Osteotomy: A Refined Surgical Technique for Medial Compartment Knee Osteoarthritis

**DOI:** 10.1002/atn2.70154

**Published:** 2026-06-14

**Authors:** Mohammad Ayati Firoozabadi, Mohammad Rastegar, Pouya Tabatabaei Irani, Rahmatullah Azimi, Armin Akbarzadeh, Seyed Mohammad Javad Mortazavi

**Affiliations:** ^1^ Joint Reconstruction Research Center Tehran University of Medical Sciences Tehran Iran; ^2^ Department of Orthopedic Surgery Imam Khomeini Hospital Complex Tehran University of Medical Sciences Tehran Iran; ^3^ Department of orthopaedic surgery Bushehr University of Medical Sciences Bushehr Iran; ^4^ Orthopedic & Rehabilitation Research Center Shiraz University of Medical Sciences Shiraz Iran; ^5^ Department of Orthopedic Surgery School of Medicine Shiraz University of Medical Sciences Shiraz Iran

## Abstract

Proximal fibular osteotomy is a minimally invasive and cost‐effective option for medial compartment knee osteoarthritis. This refined technique, performed under spinal anesthesia using Henry's posterolateral approach, involves a small incision approximately 10 cm distal to the fibular head, careful dissection through the peroneus longus plane, and osteotomy with a narrow saw blade protected by a distal osteotome. These modifications minimize soft tissue trauma and safeguard adjacent neurovascular structures. Early outcomes show pain relief, functional improvement, and correction of femorotibial angle. Immediate mobilization and rapid discharge highlight the safety, reproducibility, and clinical value of this approach.

## Graphical abstract

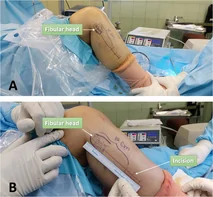

**VIDEO 1:** The patient is positioned supine, no tourniquet was used, and a Henry's posterolateral fibular approach was made with a 3‐cm skin incision, approximately 10 cm distal to the fibular head. The fibula is approached through the intermuscular plane of the peroneus longus in order to avoid peroneal nerve injury, and 1 cm was excised using an oscillating saw. The fascia is closed after proper irrigation, followed by subcutaneous tissue and skin sutures (right side). Video content can be viewed at https://doi.org/10.1002/atn2.70154.

Knee osteoarthritis (KOA) is a common degenerative joint disorder, particularly affecting elderly populations, and is frequently associated with chronic pain, restricted mobility, and reduced quality of life. Medial compartment KOA is often attributed to asymmetric load distribution during routine daily activities.^1^ Proximal fibular osteotomy (PFO) has been introduced as a minimally invasive, technically simple, and cost‐effective procedure that provides pain relief and functional improvement in patients with medial compartment KOA.^2^, ^3^ Reported benefits include shorter hospitalization, lower complication rates, and faster postoperative recovery.^4^ Although technically straightforward, PFO requires precise anatomical knowledge to avoid complications such as transient foot drop, hallux motor dysfunction, and dorsal foot paresthesia.^4^, ^5^ Multiple clinical studies have shown significant postoperative improvements in visual analog scale pain scores, Knee Society Score, and femorotibial angle correction.^6^ Despite its clinical relevance, limited technical variations of PFO have been described. To enhance nerve preservation and minimize complications, we describe a refined surgical technique.

## SURGICAL TECHNIQUE

**FIGURE 1:**
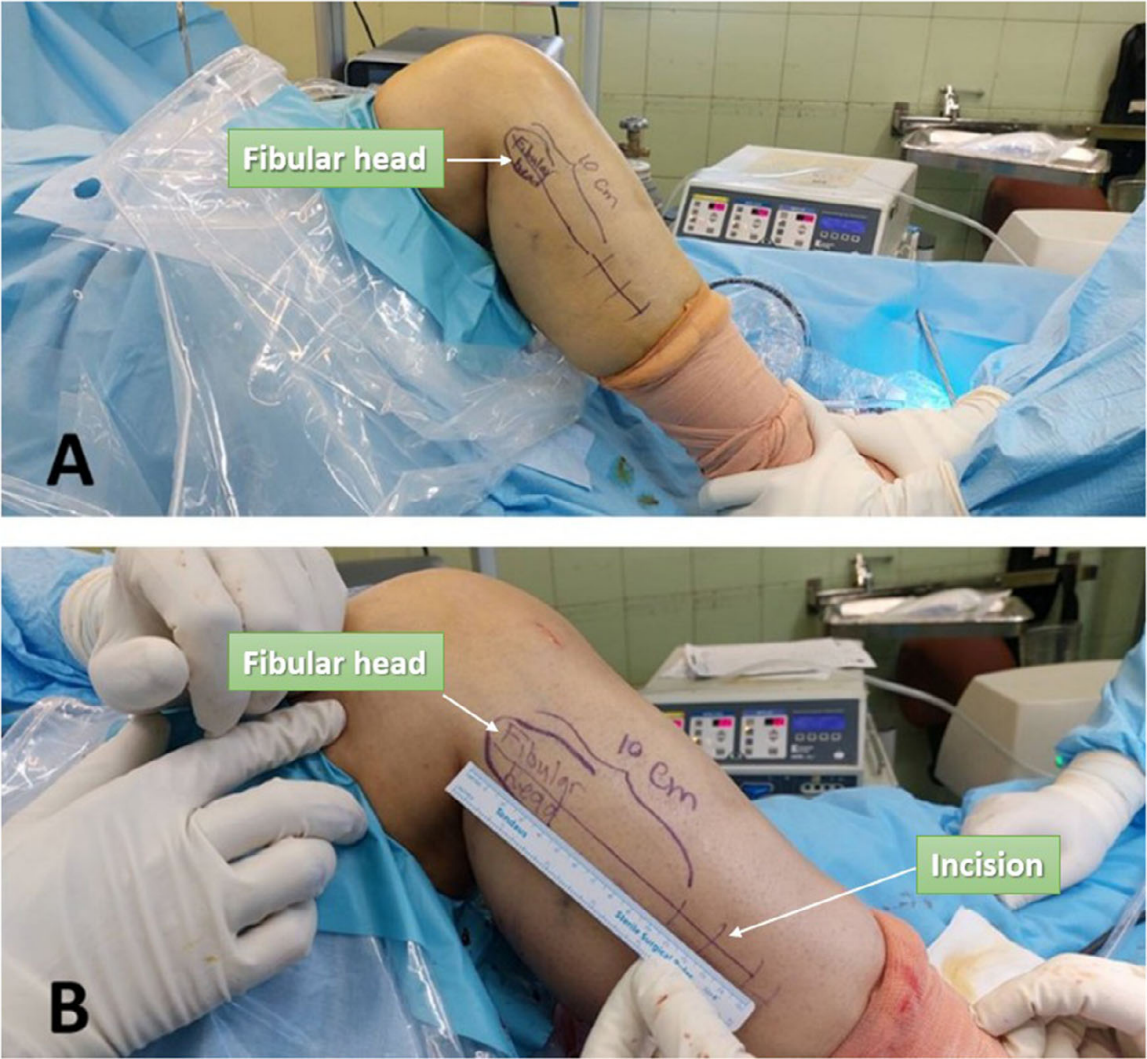
(A) Patient position and (B) skin marking before surgery (right side).

Under spinal anesthesia, the patient is positioned supine without the use of a tourniquet. Using Henry's posterolateral fibular approach, a 3‐cm skin incision is made approximately 10 cm distal to the fibular head (Figure 1).

**FIGURE 2:**
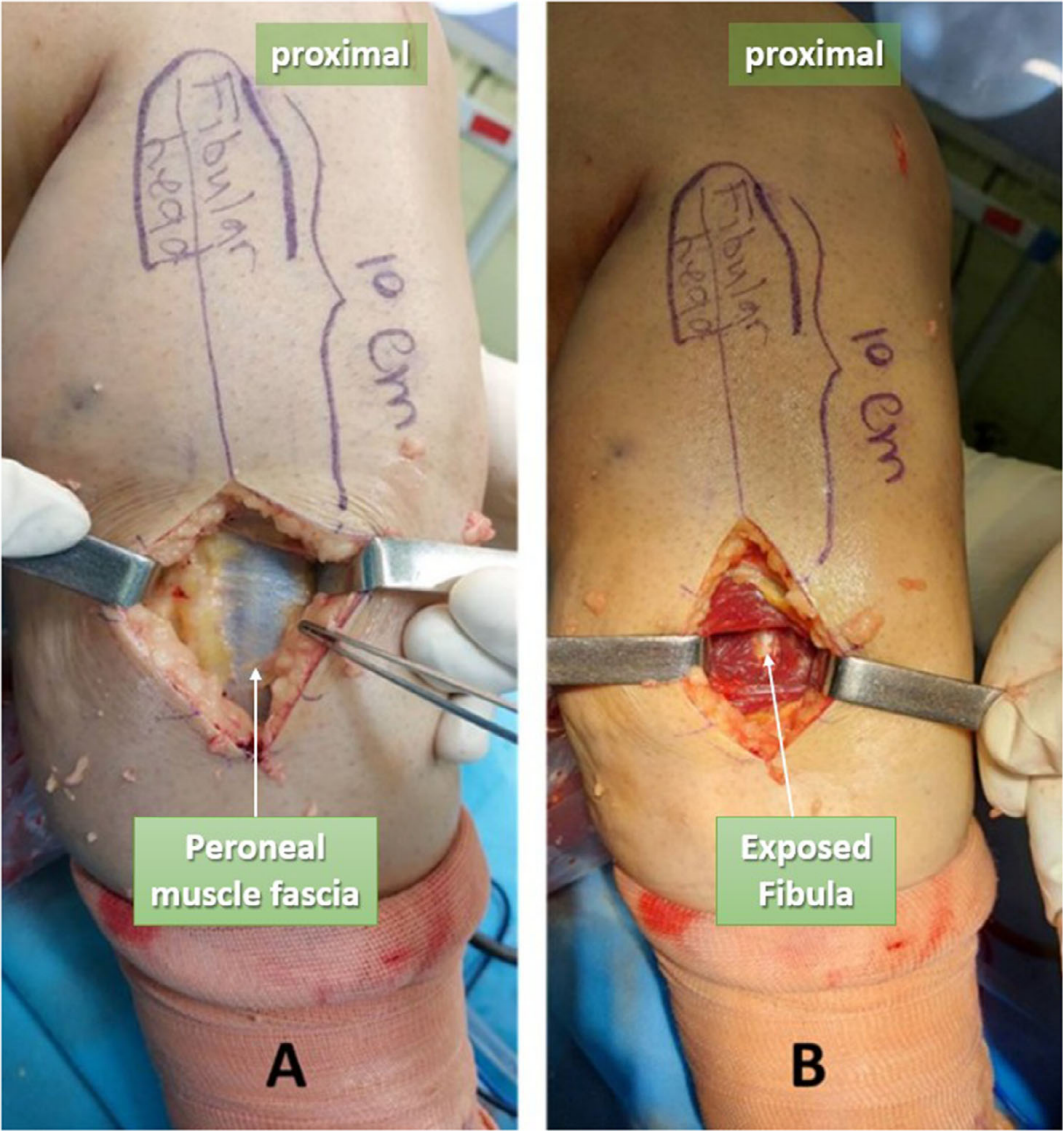
(A) Subcutaneous dissection and fascial incision exposing the muscle plane. (B) Identification of the peroneus muscle and approach to the fibula through the intermuscular plane of the peroneus longus (right side).

Subcutaneous dissection is performed, and the fascia is incised to expose the muscle plane. The peroneus muscle is identified, and the fibula is approached through the intermuscular plane of the peroneus longus (Figure 2).

**FIGURE 3:**
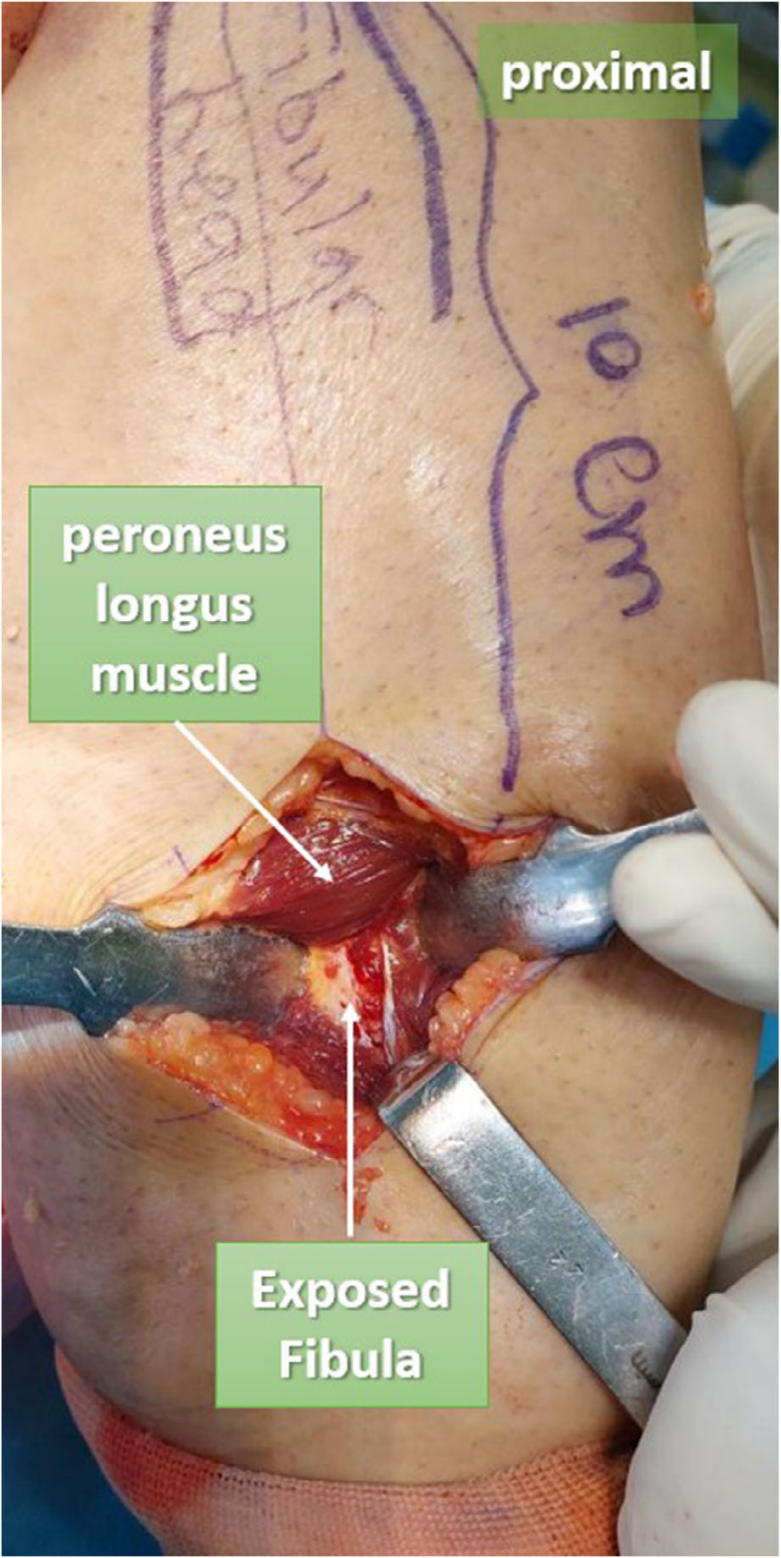
Optimal exposure of the fibula (right side).

**FIGURE 4:**
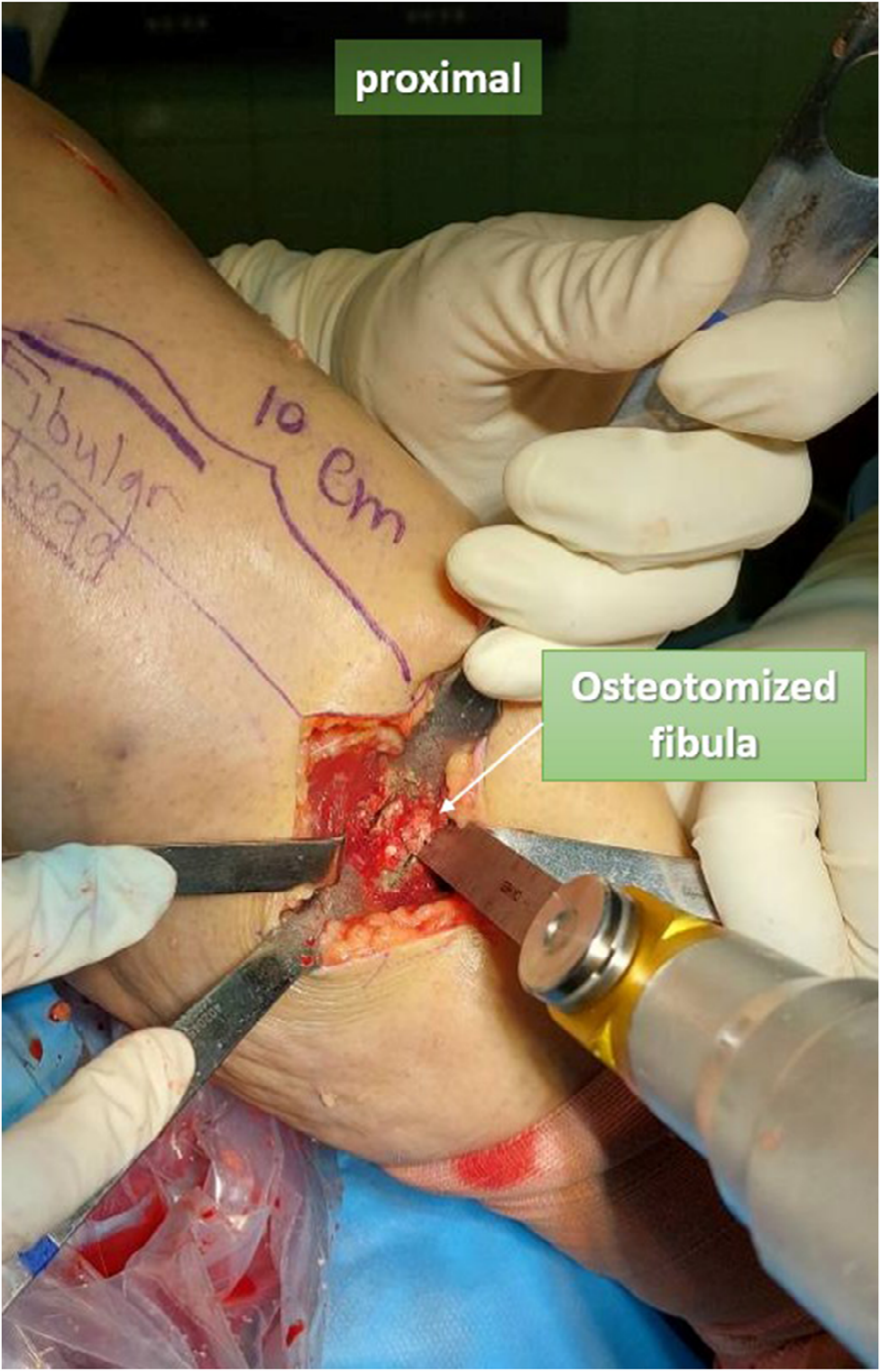
Fibular osteotomy by using an oscillating saw (Ok.med saw, Shanghai Ortho King Medical Device Co., China) (right side).

**FIGURE 5:**
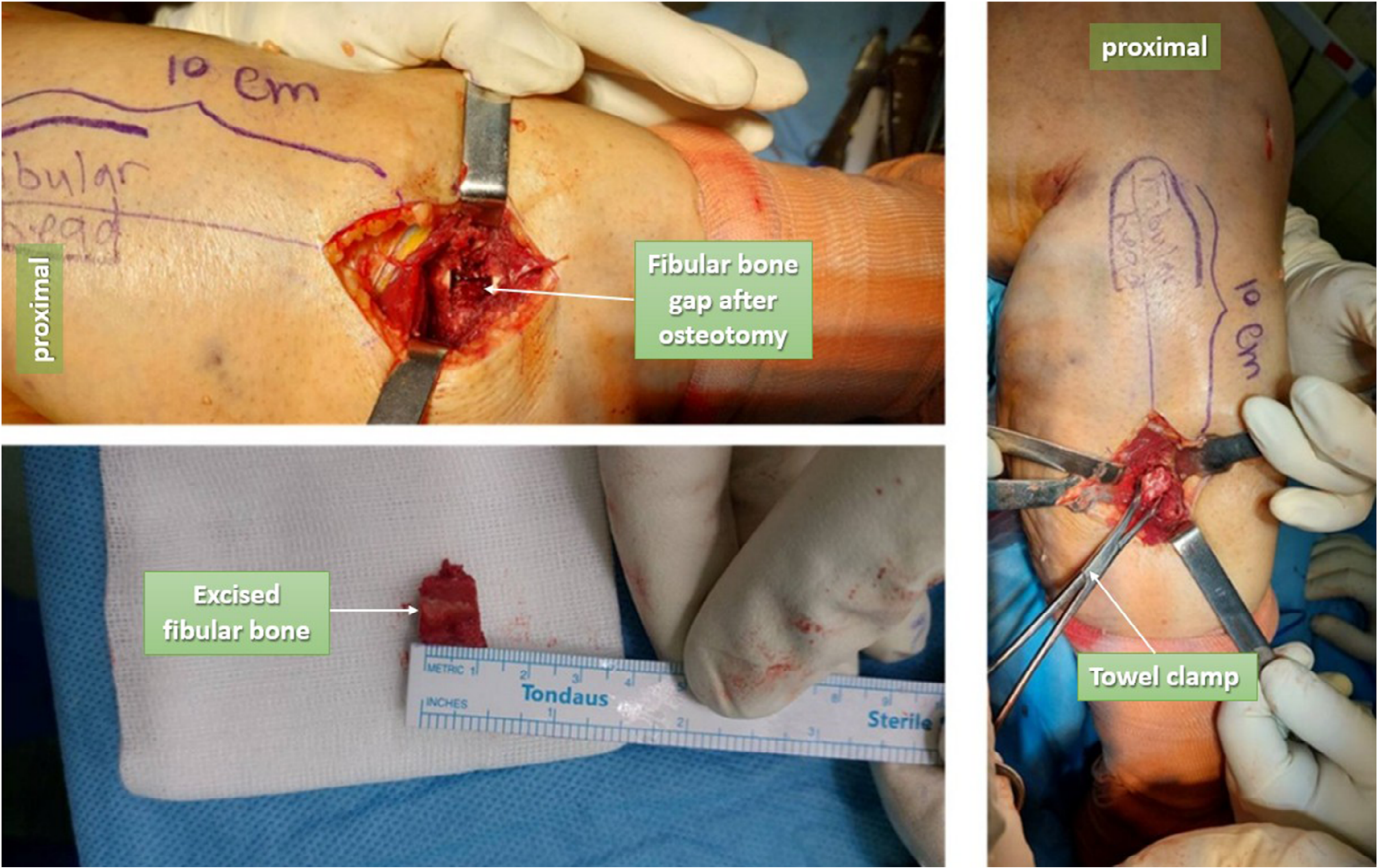
Extraction of a 1‐cm fibular segment using a towel clamp following osteotomy (right side).

Soft tissues are gently retracted with a Hohmann retractor to achieve optimal exposure of the fibula (Figure 3). An osteotomy line is marked approximately 10 cm distal to the fibular head, and the fibula is osteotomized 1 cm below the marked level using an oscillating saw (Ok.med saw, Shanghai Ortho King Medical Device Co., China) (Figure 4). To minimize injury to surrounding soft tissues and the adjacent nerve, a saw blade narrower than the fibular width is used. Additionally, an osteotome is positioned distal to the osteotomy site to shield adjacent structures and stabilize the bone during sawing. A 1‐cm fibular segment is then carefully extracted using a towel clamp (Figure 5).

**FIGURE 6:**
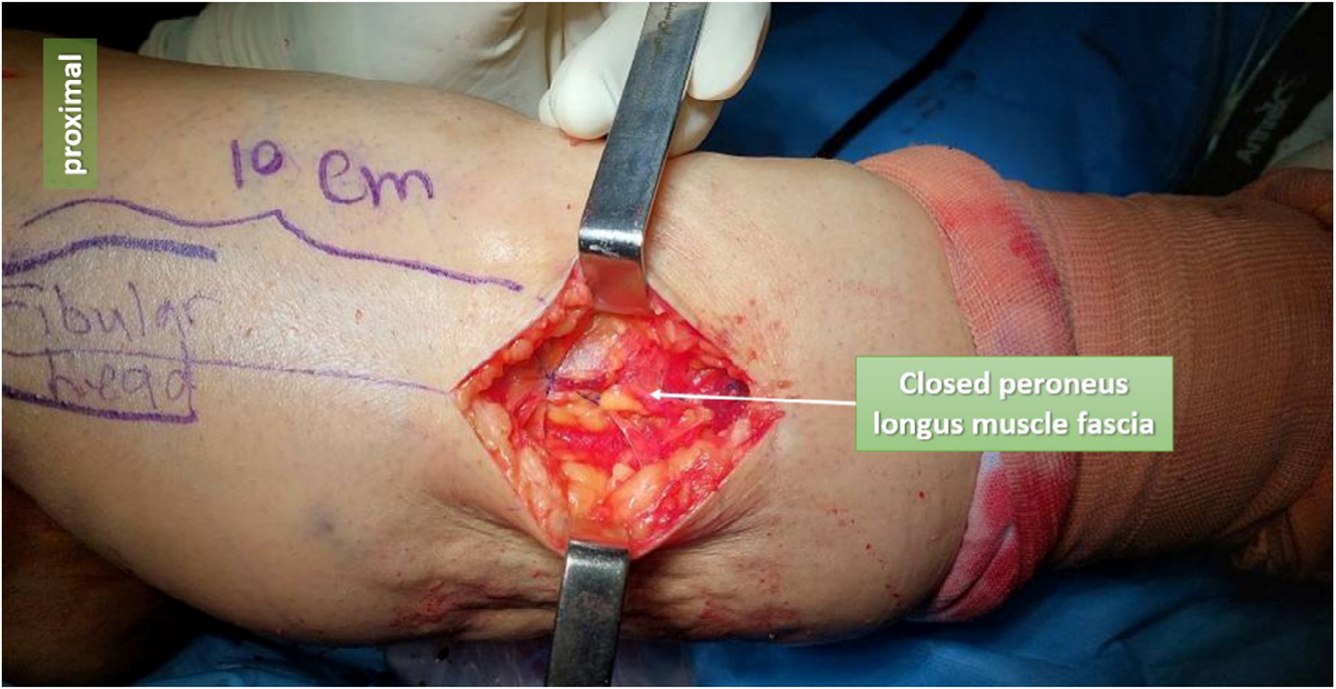
Irrigation of the osteotomy site with normal saline followed by fascial closure with sutures (right side).

The osteotomy site is thoroughly irrigated with normal saline, and the fascia is closed with sutures (Figure 6). One gram of tranexamic acid mixed with 30 mg of ketorolac is infiltrated beneath the fascial layer. The subcutaneous tissue and skin are meticulously closed with sutures. The technique is illustrated in Video 1.

**FIGURE 7:**
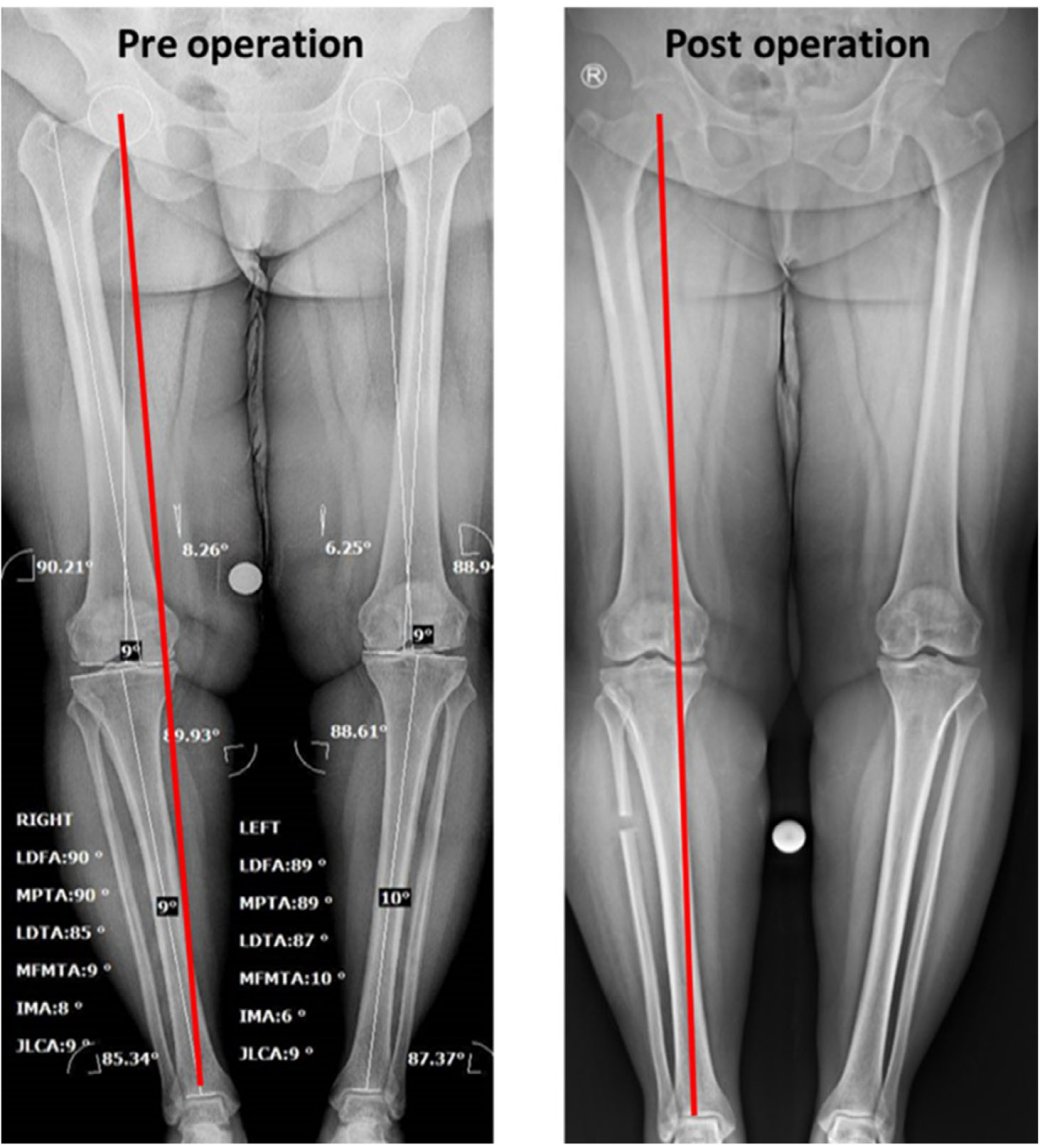
Pre‐ and postoperation of the lower limb alignment view (right side).

Pre‐ and postoperation of lower limb alignment view is shown in Figure 7. As shown in standing radiographic alignment views obtained before and after surgery, approximately 4° of lower limb varus deformity was corrected, and the bone‐on‐bone appearance of the medial tibiofemoral compartment was partially resolved.

### Post Operation Care

Immediately after anesthesia resolution, a neurovascular examination is performed, and the patient is mobilized with weight bearing as tolerated. No knee immobilization is required. Range of motion and weight bearing are initiated immediately, and the patient is discharged the following day.

## DISCUSSION

Proximal PFO represents a viable surgical option for the management of medial compartment KOA, particularly in patients with mild varus alignment and increased joint‐line congruency angle secondary to medial compartment degeneration. Clinical evidence has shown significant benefits of PFO in terms of pain reduction, functional improvement, and biomechanical realignment.^2^, ^5^, ^6^

However, the absence of standardized surgical protocols may lead to variability in outcomes and increase the risk of complications. The close anatomical relationship of the common peroneal nerve to the operative field renders it particularly vulnerable during the procedure.^4^, ^6^ The surgical technique described herein incorporates technical refinements designed to enhance the safety and reproducibility of PFO. By minimizing soft tissue disruption and providing additional protection to adjacent neurovascular structures, this approach aims to reduce complication rates and improve consistency in clinical outcomes.

To further clarify the clinical relevance of proximal PFO, the advantages and disadvantages of this procedure are summarized in Table 1. Although PFO offers several benefits such as minimal invasiveness, cost‐effectiveness, and rapid recovery, potential drawbacks, including risk of peroneal nerve injury and limited long‐term outcome data, must also be considered. This balanced overview highlights both the strengths and limitations of the technique, providing a comprehensive perspective for clinical decision‐making.

**TABLE 1:** Advantages and Disadvantages of Proximal Fibular Osteotomy (PFO)

**Disadvantages**	**Advantages**
Risk of injury to the common peroneal nerve due to anatomical proximity Limited ability to correct mechanical limb alignment Potential for an unstable fracture at the osteotomy site Limited long–term clinical evidence compared with other methods like HTO It may be inadequate for a patient with severe varus deformity	Minimally invasive approach with a small 3 cm incision and no tourniquet Enhanced soft tissue protection using a narrow saw blade and a distal osteotomy No need for implant Early weight bearing and mobilization postoperatively Effective postoperative pain management with local anesthesia and NSAIDs

HTO, high tibial osteotomy; NSAIDs, Nonsteroidal Anti Inflammatory Drugs; PFO, proximal fibular osteotomy.

Several technical pearls and pitfalls of proximal PFO are outlined in Table 2. These practical insights emphasize the importance of accurate incision placement, careful osteotomy level selection, and meticulous soft tissue handling. At the same time, potential errors such as inadequate protection of neurovascular structures or excessive retraction are highlighted. This summary provides surgeons with guidance to optimize the safety, reproducibility, and overall effectiveness of the procedure.

**TABLE 2:** Pearls and Pitfalls of Proximal Fibular Osteotomy (PFO)

**Pearls (Technical Tips)**	**Pitfalls (Potential Errors)**
Use Henry's posterolateral approach for safe access Mark osteotomy site ~10 cm distal to fibular head Employ a saw blade narrower than fibular width Place osteotome distal to osteotomy for protection Gentle soft tissue retraction with Hohmann retractor Immediate mobilization with weight bearing as tolerated	Incision too close to fibular head increases nerve risk Incorrect level may compromise biomechanical effect Wide blade may damage adjacent soft tissues Lack of shielding may injure neurovascular structures Excessive retraction can cause muscle or nerve trauma Prolonged immobilization delays recovery

PFO, proximal fibular osteotomy.

Although rare, compartment syndrome should also be considered. Preventive strategies include meticulous hemostasis, minimal muscle stripping, and gentle fascial closure. Postoperatively, patients should be observed for pain, swelling, and neurological deficits to allow for early management if necessary.

Avoidance of common peroneal nerve injury is an important point in proximal PFO due to the nerve's proximity to the fibular neck.^7^ Osteotomy performed 8‐10 cm distal to the fibular head along the intermuscular plane of the peroneus longus, with minimal soft tissue dissection, can help avoid direct nerve injury or traction neuropraxia.^8^ Other precautions include gentle retraction, proper incision placement, and the use of a thin saw blade with distal shielding to protect surrounding neurovascular structures. It must also be noted that proximal PFO is only a partial correction for lower limb deformity when compared with high tibial osteotomy. Although high tibial osteotomy is a more precise method of correcting the mechanical axis, PFO is more of a force redistribution procedure that decreases medial compartment loading through realignment, albeit not correcting the axis. Hence, PFO is generally recommended for patients with mild to moderate varus deformity and good joint mobility.

PFO offers a minimally invasive, cost‐effective, and technically straightforward option for the management of medial compartment KOA. The technique described in this article incorporates specific refinements aimed at minimizing soft tissue disruption and protecting adjacent neurovascular structures. By enhancing surgical safety and reproducibility, this approach may reduce complication rates and improve functional outcomes. PFO can therefore be considered a valuable addition to the surgical armamentarium for selected patients with medial compartment KOA, particularly those with mild varus deformity.

## DISCLOSURES

The authors (M.A.F., M.R., P.T.I., R.A., A.A., S.M.J.M.) declare that they have no known competing financial interests or personal relationships that could have appeared to influence the work reported in this article.
